# What Is the Role of Pharmacists in Treating COVID-19 Patients? The Experiences and Expectations of Front Line Medical Staff

**DOI:** 10.3389/fpubh.2021.778863

**Published:** 2021-12-20

**Authors:** Xuedong Jia, Wan Zhang, Shuzhang Du, Linlin Wen, Hongye Li, Zhao Yin, Jun Li, Xiaojian Zhang

**Affiliations:** Department of Pharmacy, The First Affiliated Hospital of Zhengzhou University, Zhengzhou, China

**Keywords:** COVID-19, pharmacists, play a role, medical staff, qualitative research

## Abstract

**Aims:** The study aimed to understand the role and the core values of pharmacists and the professional expectations of medical staff for pharmacists in treating COVID-19 patients from the perspectives of the frontline medical staff. The findings help to understand and provide a reference for the career growth path of future pharmacists.

**Methods:** A phenomenological method was used to conduct in-depth interviews with frontline medical staff working in isolation wards during COVID-19. The interview data were analyzed, and the themes were extracted.

**Results:** Pharmacists played a positive role in ensuring the supply of non-routinely stocked drugs, including traditional Chinese medicine preventative preparations, providing drug information and medication consultation for complex patients, and identifying adverse drug reactions. However, at present, the integration of pharmacists and nurses is poor with inadequate communication, and the pharmaceutical care activities provided to physicians were still not comprehensive.

**Conclusions:** The level of pharmaceutical care provided by pharmacists needs to be further strengthened. Frontline medical teams generally have high professional expectations for pharmacists, including expecting pharmacists to become drug therapy experts. They expect pharmacists to fully participate in clinical decision-making, especially playing a central role in managing drug interactions, contraindications, and other clinical uses of drugs.

## Introduction

With the deepening of China's new medical reform and proposing of the Zero Markup Drug Policy, pharmaceutical care in China is facing a significant transformation. Cognitive pharmacist services are the core direction of the future hospital pharmacy. Accordingly, establishing clinical pharmacy services has been proposed, and they have been developing rapidly in recent years, gradually narrowing the gap between China and developed countries. Academic groups and medical institutions at all levels are also exploring a pharmaceutical care model that is more suitable for China's national conditions. Remarkable achievements have been made (1–3).

The outbreak of COVID-19 at the end of 2019 has become a global pandemic (4). Medical workers have played a vital role in preventing and controlling the pandemic (5, 6). In the context of the transformation of pharmaceutical care in China, exploring the core roles that pharmacists should play in COVID has important guiding significance for the training direction of future pharmacists, unifying the core competence and formulating the evaluation system of pharmacists, and proposing directions of hospital pharmacy. To participate in all aspects of clinical treatment from pharmacists' perspectives, Chinese pharmacists have published many studies (7–17). These studies describe pharmacists' clinical activities in pharmacotherapy consultation and drug therapy monitoring, chronic disease management, and drug supply guarantee in public health crises. Researchers from Saudi Arabia explored pharmacists' non-traditional roles, and barriers and facilitators in performing these roles during the COVID-19 epidemic (18). One study examined strategies to strengthen the role of community pharmacists in public health (19). Another study discussed the impact of the COVID-19 pandemic on clinical pharmacists working in Malaysian hospitals and how clinical pharmacy is considered a health care service (20). These three qualitative studies introduced the non-traditional roles of pharmacists, the roles of pharmacists in public health, and the influence of pharmaceutical care under public health crises from pharmacists' perspectives. However, there have been no reports on the roles of pharmacists from the viewpoint of medical staff. Through qualitative research, this study aimed to explore pharmacists' roles in managing COVID-19 patients from the perspective of frontline medical staff and the professional expectations of medical staff for pharmacists. The findings help to provide important reference information for the career growth path of future pharmacists.

### Methods and Participants

The study was approved by the First Affiliated Hospital of Zhengzhou University Institutional Review Board (No.2019-KY-304). Subjects were selected from a large tertiary hospital in Henan Province (with more than 8,500 beds) using an objective sampling method. During the epidemic, the hospital is a provincial COVID-designated hospital with 35 beds for treating non-severe COVID-19 patients. The inclusion criteria of study subjects were: (1) worked in the isolation ward for more than 2 weeks for the first time, (2) directly treated COVID-19 patients, (3) had some knowledge about the work of a pharmacist, and (4) willing to participate in the study. The recruitment of study subjects continued until data saturation was reached (no new themes and information were identified during interviews).

Phenomenological research methods were adopted to conduct one-on-one in-depth semi-structured interviews with the research objects. The research team developed an interview outline based on the research purpose, literature reports, and practical experiences. The interview primarily focused on two questions: (1) what positive roles did Chinese pharmacists play in fighting against COVID-19? (2) what kind of help did you want pharmacists to provide in the future? A more detailed interview guideline is presented as Supplementary Material. The interview guide was tested with two study subjects and revised for clarity before starting the study.

### Data Collection Methods

The researcher (XD J) contacted the interviewees by phone or email and explained the study to determine whether they were willing to participate in the study. Some refused to participate in this study because they were too busy with work or did not want to recall their painful experiences in fighting the epidemic. After the respondents signed written informed consent, the interview was arranged in a comfortable environment, usually in the pharmacist's office or the clinician's office. The interview was recorded. Changes in the emotions of the subjects and their body movements were noted during the interview. Interviews usually lasted 15–30 min. The study was conducted from December 2020 to May 2021.

### Data Analysis Methods

Within 24 h after the end of each interview, the researchers sorted out the audio recordings of the interviews and transcribed them into written materials. Data were managed and analyzed using NVivo12 software. The Colaizzi seven-step analysis method [(1) Transcribing all descriptions; (2) Extracting significant statements; (3) Creating Formulated meaning; (4) Building themes; Developing an exhaustive desciption; (5) Identifying the fundamental structure of the phenomenon; (6) Returning to participants for validation (21)] was utilized. Two researchers independently completed the data extraction and obtained the initial theme codes. Two team members discussed the codes and drafted the themes and sub-themes. Discrepancies were discussed among team members to reach a consensus. Representative quotations were selected. Data were reported under the guidance of the Consolidated Criteria for Reporting Qualitative Studies (COREQ) Checklist (22) (Supplementary Material).

### Integrity

To ensure integrity of the research, all researchers received systematic training on qualitative research methodology and mastered interviewing skills and data analysis methods. The transcripts were sent to the interviewees to confirm the authenticity and integrity of the contents.

## Results

A total of 11 respondents (eight doctors and three nurses; seven females and four males) participated in the study (Table 1).

**Table 1:** Subject's basic information (*n* = 11).

**No**.	**Age (years)**	**Work experience (years)**	**Days worked in the COVID-19 ward**	**Title**	**Department**
D1	49	29	22	Chief Physician	EICU
D2	49	22	22	Surgeon-in-charge	EICU
D3	33	7	22	Surgeon-in-charge	RICU
D4	29	3	22	Surgeon-in-charge	ICU
D5	30	2	22	Surgeon-in-charge	ICU
D6	38	15	22	Chief Physician	Respirology
D7	33	5	14	Doctor-in-charge	Cardiovasology
D8	30	2	14	Doctor-in-charge	Hematology
N1	33	7	21	Nurse-in-charge	Thoracic surgery
N2	34	6	22	Chief Nurse	PICU
N3	31	6	21	Senior Nurse	PICU

### Theme 1. Pharmacists Play an Active Role in COVID-19 Prevention and Control

#### Theme 1.1-Provide Hospital Preparations for Medical Staff in Epidemic Prevention and Control

During the COVID-19 epidemic, the Traditional Chinese Medicine (TCM) dispensing room developed TCM preparations based on TCM theories in preventing infectious diseases and sent them to the frontline medical staff. These preparations aimed to relieve anxiety and loss of appetite, and improve immunity, which was well received by the frontline health care workers.

*N1: “I think you (pharmacists) are very good. We drank the Chinese medicines you made, and our team members all drank them fast (high spirits)... And they all felt it was very good.”*

*N3: “The Chinese medicine, the Epidemic Prevention Three, was sent to the isolation ward. Maybe this medicine was a spiritual pillar for everyone. I drank it, and all of you look at it, and I can increase resistance. You are OK, and I can rest assured, with great faith in everyone.”*

*D7*: “*I think the most impressive thing is Chinese medicines for prevention, and also we sometimes drink the Chinese medicine One, Two, and Three preparations sent by the pharmacy, and I think it is quite good.”*

#### Theme 1.2-Provide Essential Drug Information and Ensure Drug Supply

Pharmacists have played an active role in ensuring the supply of non-routinely stocked medicines in hospitals during the COVID-19 pandemic and providing information for these drugs.

*D3: “We used a drug that we had never used before that is called XX from the pharmacy department, and also they sent us the data and instructions about the use of the drug, adverse reactions, and how to prepare the drug in detail, which was very helpful to us.”*

*D6: “The pharmacist has done a great job when my patient wants to use a new drug or the one that is not currently stocked in the hospital. They can get it within hours basically. We always encountered pharmacists or nurses who were sacrificing their own time to procure the drugs.”*

#### Theme 1.3-Participate in the Consultation and Handle the Rationality of the Medication Plan

Pharmacists participated in the consultation of the clinical treatment plan and parenteral nutrition and played an active role in controlling the rationality of the medication plan and promoting the nutritional rehabilitation of patients.

*D1: “The pharmacist will participate in the hospital teleconsultation which often controls the direction and rational use of medications. It is also a good thing.”*

*D6: “After the discussion of respiratory issues and infections, the pharmacists will evaluate whether the drug can be used like this. And we also communicate with the pharmacists about the time, the response, the dosage, and the subsequent response of the patient to the drug, which I think can help a lot (high spirits).”*

*D8: “Nutritional specialty pharmacists provide daily consultations to patients on how to get a good supplement of sugar, protein, and lipid. They play a good supporting role in this aspect.”*

#### Theme 1.4-Guidance on Contraindications, Drug Interactions, and Adverse Drug Reactions

In terms of clinical problems that pharmacists participated in, physicians reflected that pharmacists played an essential role in the judgment of drug interactions, the identification of contraindications, and assessing adverse drug reactions. Doctors believed that pharmacists had more professional advantages and could complement clinicians to determine the safety and rationality of the dosage schedule.

*D1: “We can't recognize the adverse reactions, especially the contraindications, while they (pharmacists) can. They may think more of the interactions of drugs and whether the drugs are metabolized by the liver enzymes or not, which is good. The pharmacists are excellent, as a matter of fact.”*

*D3: “They can judge some side effects of antiviral drugs. Some patients had serious gastrointestinal reactions after taking them, and then they consulted with clinical pharmacists to find the side effects of this drug, which was very important for us to adjust the regimen.”*

*D4: “If a pharmacist goes to a consultation, they will focus on drug interactions and whether the drugs can be used together.”*

*D7: “Pharmacists can give us a guide for things such as contraindications and drug interactions, which we don't consider so comprehensively sometimes.”*

### Theme 2. Career Expectations of Medical Staff for Pharmacists in the Future

Medical teams generally have high professional expectations for pharmacists. They expect pharmacists to be drug treatment experts who can put forward constructive opinions and play a fundamental guiding role in controlling drug use. They also have great expectations for the professional value of pharmacists.

*D2: “As far as I know, those hospitals in Beijing and Shanghai are equipped with clinical pharmacists who are specifically responsible for supervising the standardization and rationality of drug use in their departments, which is very good.”*

*D6: “In the future, if there is an expert consultation, I suggest a clinical pharmacist can participate in it. Although every team can't have its pharmacist, a clinical pharmacist must be added during the expert consultation so that he will play a role in hammering the rationality of medication usage.”*

*N2: “For example, as a nurse, I often can't sleep at night., so I take a Stilnox from the pharmacy. Can you give us a hint about how to take this medicine? Is it half a pill at once? How to take it? I think it is necessary to get some medication guidance and feedback from the frontline personnel on the effect of medication with the help of the pharmacist.”*

#### Theme 2.1-Pharmacists Are Expected to Fully Participate in the Clinical Team

The medical team expects pharmacists, as members of the clinical multidisciplinary team, to fully participate in the team and make decisions on clinical issues in the future.

*D5: “Pharmacists can help us to identify whether this medicine is useful for this patient. We can communicate more frequently with the pharmacists about the dose or the drug efficacy, and then we assess the clinical effect of the drugs together.”*

*N2: “I think the pharmacist provides some pharmaceutical care advice to patients and the medical staff. I don't know whether that's the pharmacist's job to keep track of the medical staff as a part of the team (laughter)”*

#### Theme 2.2-Pharmacists Assess Drug Interactions and Complex Medication Regimens

If the pharmacists can participate, the medical team expects pharmacists to identify drug interactions and adverse drug reactions and assess the rationality of drug use regimens. Therefore, pharmacists should strengthen their professional knowledge in the future.

*D3: “I think doctors may not have enough knowledge in the aspect of drug interactions, or we may not think what the possible effects between the two drugs are after using different kinds of drugs. I hope the pharmacist gives us a reminder and guidance.”*

*D4: “We hope that the pharmacist will be able to give us a little bit of advice on the basic information of this drug, the interactions, and the compatibility between drugs.”*

*D8: “We pay attention to a phenomenon in which hydroxychloroquine and azithromycin are used together in which they actually both have a strong effect on the QT prolongation. We hope that if we have a similar situation next time, the pharmacist could provide some guidance from the perspective of clinical treatment.”*

*N2: “We expect a pharmacist to remind you about what you can't take when you finish your drug or what side effects you need to watch out for after you take the drug.”*

### Theme 3 Deficiencies in Pharmacists' Pharmaceutical Care

#### Theme 3.1-Pharmacists Did Not Make Real-Time Interventions

The results of this interview also show that some doctors think that pharmacists did not intervene in time. There is less communication with pharmacists on epidemic prevention and control as they do not know what help pharmacists can provide. If pharmacists can make real-time interventions, they may play a more significant professional role.

*D1: “I think it would have been better if the pharmacist had intervened earlier, or if the pharmacist often gave some advice in the early stage.”*

*D2: “It would be positive if there were pharmacists in the team, but right now, our team is lacking pharmacists*.

*D4: “There was little contact with pharmacists, and we basically discussed our issues with other clinical experts.”*

#### Theme 3.2-Less Communication Between Pharmacists and Nursing Staff

This study shows that the nursing team generally reported less contact with pharmacists. The nursing staff has little understanding of the pharmacists, which is limited to the professional image of drug dispensing. In the future, pharmacists need to strengthen the integration with the nursing team to promote communication.

N1: “*I* haven't really thought about the pharmacists because I don't know what they can do for us. We go to the doctor directly if there is a problem. A doctor is in charge of a patient, so we will contact the doctor directly. If there is a problem, the doctor will contact the pharmacist.”

*N2: “The pharmacist did not come to the front line this time. We have less contact at work.”*

*N3: “I do not know the pharmacist, and also it seems that we have no knowledge on the help the pharmacist can provide.”*

## Discussion

From the perspectives of medical staff, this study shows that pharmacists played a positive role in ensuring the supply of non-routinely stocked drugs such as TCM preventative preparations, providing drug information and medication consultation, and identifying adverse drug reactions and interactions during the COVID epidemic prevention and control. However, the integration between pharmacists and the nursing team is poor, with less communication. Pharmaceutical care provided by pharmacists is not comprehensive enough to cover the needs of doctors, which needs to be further strengthened in the future. Medical teams generally have high professional expectations for pharmacists. To become drug treatment experts in the future, pharmacists should fully participate in the clinical work and the decision-making of clinical issues and play a central role, especially in the judgment of drug interactions and contraindications.

With the deepening of China's new medical reform and the transformation of pharmaceutical care, pharmacists play an increasingly important role in patients' care. Many publications have been published describing various pharmaceutical care activities provided by pharmacists (7–17). Example activities are providing drug therapy monitoring and patient medication education, assessing adverse drug reactions, optimizing medication plans for special population patients, participating in clinical medication plan decision-making, providing medication consultation to physicians and nurses, and managing patients with chronic diseases through medication therapy management. However, this study shows that the medical team still has a significant lack of understanding of pharmacists and the professional help that pharmacists can provide. At present, the knowledge of the core role of pharmacists is still focused on the judgment of adverse drug reactions, the discrimination of drug interactions, and the guarantee of drug supply with less understanding of other work that pharmacists can contribute.

The Saudi Arabian qualitative study showed that the acceptance of pharmacists' non-traditional roles was generally positive at the policy level (18). However, the acceptance of these non-traditional roles by other healthcare professionals and patients required further education. For future professional development and the role of Chinese pharmacists, we need to make efforts in several aspects. The government needs to provide support at the policy level for the clinical status of pharmacists to promote the enthusiasm and acceptance of pharmacists. Europe, Canada, the United States, and other developed countries have given more privileges to pharmacists in various degrees during the COVID-19 pandemic to actively participate in patients' care (23). Pharmacists should further strengthen the publicity and education of the medical team. They should actively participate in the clinical work to help the medical team better understand the pharmaceutical care abilities of pharmacists. A scoping review of published studies suggests that future research with a more detailed description and an evaluation of the impact of pharmacist intervention is needed to guide future actions in COVID and/or other pandemics (24), which is an opinion confirmed by our study. Another study explored strategies to strengthen the role of community pharmacists in public health by using qualitative research (19). The study found that enhancing public health training for pharmacists and teaching communication methods to pharmacists might be effective strategies to strengthen the role of community pharmacists in public health.

Our study shows that pharmacists did not fully participate in COVID diagnosis and treatment from the perspectives of medical staff during the outbreak. In addition, pharmacists did not directly participate in bedside pharmaceutical care. In many cases, the pharmaceutical care work was provided because the medical team actively invited pharmacists to participate, and pharmacists did not participate proactively. The findings are similar to a qualitative study conducted in Malaysia (20). In this study, Chinese pharmacists participated in treating COVID-19 patients through telemedicine. However, there is a gap between these services and the needs of the medical team. Therefore, in the future, society and other medical workers should give more significant support and recognition to the role of pharmacists. Pharmacists should also enhance their integration with medical staff to provide better care to patients.

Our study shows that the medical team has high professional expectations for pharmacists. They think that a pharmacist should be a drug therapy expert and be competent enough to solve various problems related to drug therapies. At present, Chinese clinical pharmacy has been developing gradually toward specialization, and the training of clinical pharmacists is also divided into different subject areas. However, the expectation from the medical team is that the pharmacist should be a drug information expert and a pharmacotherapy expert who can comprehensively evaluate the medication plan. Given these findings, the training of pharmacists in the future should focus on both specialty and general training. A well-rounded general practice clinical pharmacist with expertise in a specific field may be more adaptable to the clinical expectations for pharmacists.

The study has several limitations. It is a single-center study, and the results may not be generalizable to describe the pharmaceutical care provided by the overall Chinese pharmacist profession. The study also has a small sample size.

This study shows that during the COVID-19 outbreak, Chinese pharmacists expanded their professional roles. Pharmacists developed TCM preparations for frontline healthcare providers based on the needs of these professionals. These TCM preparations played essential roles in relieving medical workers' anxiety and improving their immunity. When fighting against the epidemic, frontline medical workers used medications to treat their symptoms, such as insomnia. The pharmacist then guided the choice of treatment drugs. In addition, pharmacists actively provided consulting services on the proper use of new therapeutic drugs for COVID, including off-label use. These expanded roles have been positively recognized by the frontline medical staff. However, medical staff have also expressed more expectations for pharmacists. The competencies of pharmacists need to be further strengthened to meet the needs of the medical team.

## Data Availability Statement

The original contributions presented in the study are included in the article/Supplementary Material, further inquiries can be directed to the corresponding authors.

## Ethics Statement

The studies involving human participants were reviewed and approved by First Affiliated Hospital of Zhengzhou University Institutional Review Board. The patients/participants provided their written informed consent to participate in this study (No.2019-KY-304).

## Author Contributions

XZ, ZY, and XJ: conception and design. XZ, JL, and SD: administrative support. XJ, ZY, WZ, HL, and LW: collection and assembly of data. XJ, ZY, WZ, HL, LW, and XZ: data analysis and interpretation. XJ, ZY, and XZ: manuscript writing. XJ, WZ, SD, LW, HL, ZY, JL, and XZ: final approval of manuscript. All authors contributed to the article and approved the submitted version.

## Conflict of Interest

The authors declare that the research was conducted in the absence of any commercial or financial relationships that could be construed as a potential conflict of interest.

## Publisher's Note

All claims expressed in this article are solely those of the authors and do not necessarily represent those of their affiliated organizations, or those of the publisher, the editors and the reviewers. Any product that may be evaluated in this article, or claim that may be made by its manufacturer, is not guaranteed or endorsed by the publisher.
