# Supplementary material for: What Is the Role of Pharmacists in Treating COVID-19 Patients? The Experiences and Expectations of Front Line Medical Staff
Source: Front Public Health. 2021 Dec 20;9:778863. doi: 10.3389/fpubh.2021.778863 (PMC8720774; doi:10.3389/fpubh.2021.778863)
Supplement: Supplementary file 1 [file Data_Sheet_1.docx]

**S1：Questions used in the interview guide**

Opening questions:

1. In the prevention and control of this epidemic, did you feel that there are pharmacists around you playing a positive role?
2. During the prevention and control of this epidemic, what problems did you encounter related to medication treatment?

Q1 What positive role did medicine pharmacists play in fighting against COVID-19?

1. In the early stage, there were participants mentioned the confusion of off-label medication. What role did the pharmacist play in this regard?

Q2 What kind of help did you want pharmacists to provide in the future?

1. Did you have any confusions when preparing new drugs?
2. Did you want pharmacists to participate in the consultation? In what areas do you want them to give advice?

**S2--COREQ for reporting qualitative studies**

**The checklist of the research**

Consolidated criteria for reporting qualitative studies (COREQ): 32-item checklist

| No Item | Guide questions/description | Answers |
| --- | --- | --- |
| **Domain 1: Research team and reflexivity** | | |
| Personal Characteristics | | |
| 1. Interviewer/facilitator | Which author/s conducted the interview or focus group? | Xuedong Jia and Zhao Yin |
| 2. Credentials | What were the researcher’s credentials? E.g. PhD, MD | MD (Zhao Yin) or MD (Xuedong Jia) |
| 3. Occupation | What was their occupation at the time of the study? | Pharmacist |
| 4. Gender | Was the researcher male or female? | Male (Xuedong Jia) and Male (Zhao Yin) |
| 5. Experience and training | What experience or training did the researcher have? | Theoretical training and experience of conducting several qualitative studies with other groups. |
| Relationship with participants | | |
| 6. Relationship established | Was a relationship established prior to study commencement? | Yes |
| 7. Participant knowledge of the interviewer | What did the participants know about the researcher? e.g. personal goals, reasons for doing the research | Reasons for doing the research |
| 8. Interviewer characteristics | What characteristics were reported about the interviewer/facilitator? e.g. Bias, assumptions, reasons and interests in the research topic | Reasons and interests in the research topic |
| **Domain 2: study design** | | |
| Theoretical framework | | |
| 9. Methodological orientation and Theory | What methodological orientation was stated to underpin the study? e.g. grounded theory, discourse analysis, ethnography, phenomenology, content analysis | Phenomenology. |
| Participant selection | | |
| 10. Sampling | How were participants selected? e.g. purposive, convenience, consecutive, snowball | Purposive and convenience. |
| 11. Method of approach | How were participants approached? e.g. face-to-face, telephone, mail, email | Face-to-face interview |
| 12. Sample size | How many participants were in the study? | 11 |
| 13. Non-participation | How many people refused to participate or dropped out? Reasons? | Three, they were too busy with work or they did not want to recall their painful experiences in fighting the epidemic |
| Setting | | |
| 14. Setting of data collection | Where was the data collected? e.g. home, clinic, workplace | Workplace. |
| 15. Presence of non-participants | Was anyone else present besides the participants and researchers? | No |
| 16. Description of sample | What are the important characteristics of the sample? e.g. demographic data, date | Yes, demographic data. |
| Data collection | | |
| 17. Interview guide | Were questions, prompts, guides provided by the authors? Was it pilot tested? | Yes |
| 18. Repeat interviews | Were repeat interviews carried out? If yes, how many? | No |
| 19. Audio/visual | Did the research use audio or visual recording to collect the data? | Audio recording |
| 20. Field notes | Were field notes made during and/or after the interview or focus group? | Yes |
| 21. Duration | What was the duration of the interviews or focus group? | 15-30 min |
| 22. Data saturation | Was data saturation discussed? | Yes |
| 23. Transcripts returned | Were transcripts returned to participants for comment and/or correction? | Yes |
| **Domain 3: analysis and findingsz** | | |
| Data analysis | | |
| 24. Number of data coders | How many data coders coded the data? | Two |
| 25. Description of the coding tree | Did authors provide a description of the coding tree? | No |
| 26. Derivation of themes | Were themes identified in advance or derived from the data? | Yes |
| 27. Software | What software, if applicable, was used to manage the data? | NVIVO 12 |
| 28. Participant checking | Did participants provide feedback on the findings? | Yes |
| Reporting | | |
| 29. Quotations presented | Were participant quotations presented to illustrate the themes / findings? Was each quotation identified? e.g. participant number | Yes |
| 30. Data and findings consistent | Was there consistency between the data presented and the findings? | Yes |
| 31. Clarity of major themes | Were major themes clearly presented in the findings? | Yes |
| 32. Clarity of minor themes | Is there a description of diverse cases or discussion of minor themes? | Yes |
